# Supplementary material for: nextPYP: a comprehensive and scalable platform for characterizing protein variability in situ using single-particle cryo-electron tomography
Source: Nat Methods. 2023 Oct 26;20(12):1909–19. doi: 10.1038/s41592-023-02045-0 (PMC10703682; doi:10.1038/s41592-023-02045-0)
Supplement: Supplementary file 1 — Supplementary Fig. 1 and Supplementary Tables 1–5. [file 41592_2023_2045_MOESM1_ESM.pdf]

nature methods

Article https://doi.org/10.1038/s41592-023-02045-0

nextPYP: a comprehensive and scalable platform for characterizing protein variability in situ using single-particle cryo-electron tomography

In the format provided by the authors and unedited

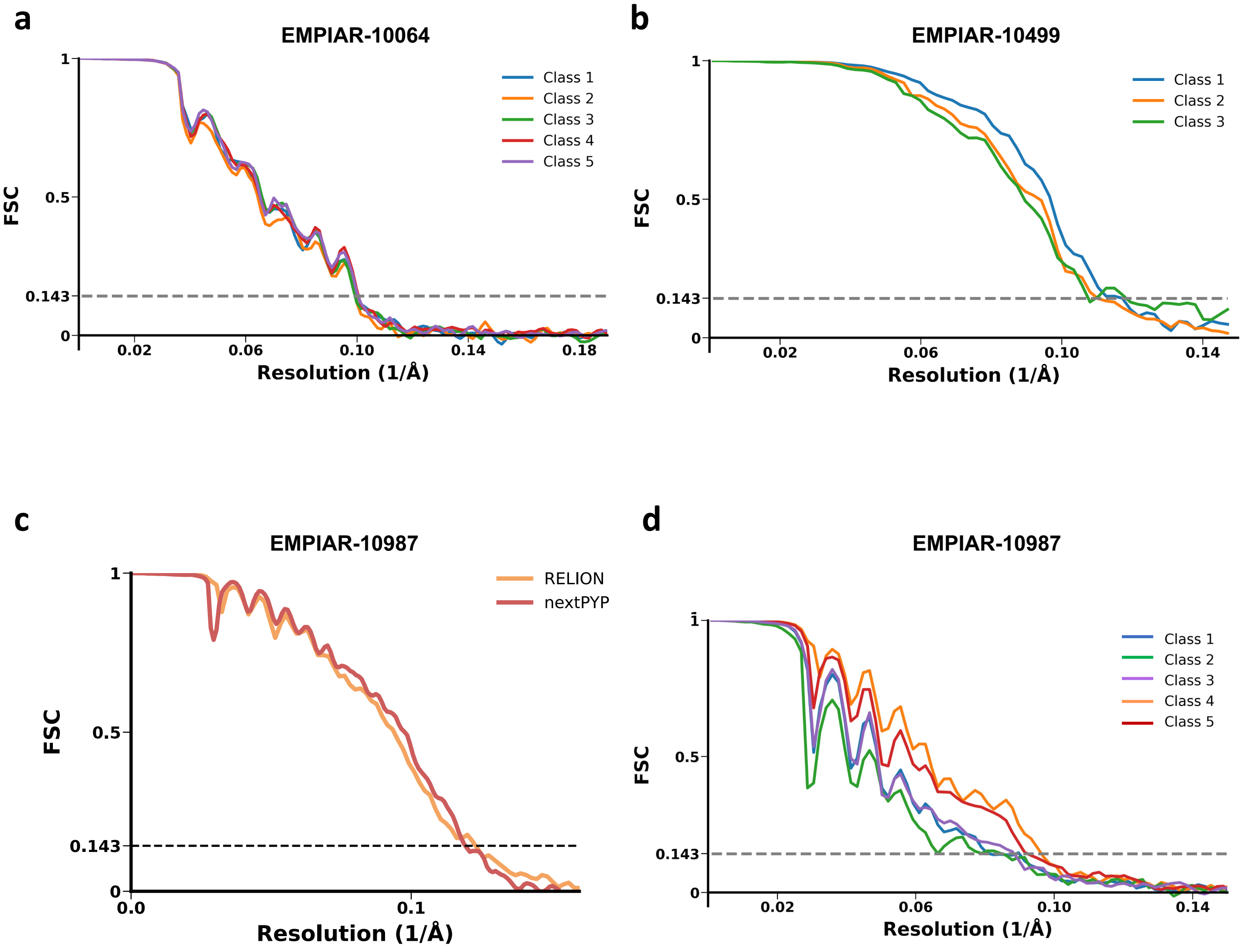

Supplementary Fig. 1 | Fourier Shell Correlation (FSC) plots for ribosome datasets analyzed by nextPYP.
a) In-vitro 80S ribosomes from EMPIAR-10064. 5 classes were obtained with resolutions of 10.0 Å, 10.2 Å, 10.2 Å, 10.0 Å, and 10.0 Å, respectively. **b)** Native ribosomes from intact M. pneumoniae cells from EMPIAR-10499. 3 classes were obtained with resolutions of 8.7 Å, 9.3 Å, and 9.5 Å, respectively. **c)** Consensus map of 80S ribosome from in-situ lamellae from EMPIAR-10987 using RELION (8.2 Å, 9,447 particles) and nextPYP (8.4 Å, 6,763 particles). **d)** Classification of EMPIAR-10987 into 5 classes with resolutions of 12.4 Å , 11.8 Å , 11.4 Å, 10.5 Å , and 10.9 Å, respectively.

PACKAGE NAME	EMAN2 [38]	emClarity [35]	Dynamo [30]	Relion-4.0 [36]	tomoBEAR/ SUSAN [27]	Warp/M [26], [37]	nextPYP [This work]
**Pre-processing**							
Movie-frame alignment	No	No	No	No	Yes (live)	Yes (live)	Yes (live)
Tilted CTF estimation	Yes	Yes	Yes	No	Yes (live)	Yes (live)	Yes (live)
Tilt-series alignment	Yes	Yes	Yes	No	Yes (live)	No	Yes (live)
Tomogram generation	Yes	Yes	Yes	No	Yes (live)	No	Yes (live)
**Particle picking**							
Template-matching	Yes	Yes	Yes	No	Yes	Yes	Yes (live)[1]
Size-based	No	No	No	No	No	No	Yes (live)
Geometry-based	Yes	No	Yes	No	No	No	Yes (live)
Neural-network based	Yes[2]	No	No	No	No	No	Yes (live)[3]
**3D refinement**							
Bypass sub-volumes	No	No	No	No	Yes	No	Yes
Particle-based CTF	No	No	No	Yes	No	Yes	Yes
Movie refinement	No	No	No	No	No	Yes	Yes
3D classification	Yes	Yes	Yes	Yes	Yes	No	Yes
**Platform**							
Operating system(s)	Linux/OSX	Linux	Linux/OSX	Linux	Linux	Windows	Linux
Language(s)	Python/C++	Matlab/C	Matlab	C++/Python	Matlab/C++	C#/C++	Python/C++/Kotlin
MPI/SLURM support	Yes	No	Yes	Yes	Yes	No	Yes
CPU/GPU support	CPU/GPU	GPU only	CPU/GPU	CPU/GPU	GPU only	GPU only	CPU/GPU[4]
Graphical user interface	QT/OpenGL	No	Matlab	X11/FLTK	No	WPF .NET	Web-based
3D visualization	Yes	No	Yes	No	No	No	Yes
Import/export functions	No	No	Yes	Yes	Yes	Yes	Yes

[1] Surface-constrained template-matching
[2] Fully-supervised particle picking
[3] Semi or semi-supervised particle picking
[4] GPU is used only for training neural networks during particle picking

Supplementary Table 1. Comparison of packages for SP-CET data analysis. Features, requirements and characteristics of existing software packages for single-particle cryo-electron tomography.

DATASET	EMPIAR-10164	EMPIAR-10064	EMPIAR-10499
**Data size**			
Image size (width × height × tilts × frames)	7420×7676×41×8/10	3710×3710×59×1	3838×3710×41×12
Number of tilt-series	5	4 (defocus data)	65
Average number of particles per tilt-series	2,896	1,096	279
Box size (voxels)	384	256	256
**Storage savings per dataset**			
Storage for sub-volumes	3.0 TB	273.6 GB	141.7 GB
Storage for particle/frame stacks	2.3 TB	56.4 GB	45.1 GB
**Pre-processing time per tilt-series (mm:ss)**			
Frame alignment and averaging	03:47	N/A	03:24
Tilt-series alignment	03:54	02:40	01:35
CTF estimation and tomogram reconstruction	02:26	01:32	01:18
Particle picking	04:25[1]	00:37[2]	04:03[3]
Bookkeeping and images for visualization	01:40	01:10	01:41
**Total pre-processing time per tilt-series**	**16:12**	**05:59**	**12:01**
**Refinement time per tilt-series (hh:mm:ss | bin)**			
Reference-based refinement	02:28:03 | 4x	02:58:24 | 2x	01:38:54 | 2x
Fully constrained refinement (per iteration)	00:56:24 | 2x	00:09:07 | 1x	00:04:53 | 1x
Region-based refinement (per iteration)	00:54:18 | 2x	00:07:03 | 1x	00:04:13 | 1x
Particle-based CTF refinement	01:47:42 | 2x	00:13:09 | 1x	00:06:51 | 1x
Movie-frame refinement	02:11:24 | 2x	N/A	00:11:26 | 1x
3D classification (per iteration)	N/A	00:05:48 | 1x	00:01:23 | 1 x
**Total refinement time per-tilt series**	**08:17:51**	**03:33:31**	**02:07:40**

[1] Geometry-based picking

[2] Size-based picking

[3] Neural-network based picking (inference only, running on CPU)

Supplementary Table 2. Storage and timing statistics. Processing times are reported on a per tilt-series basis. One virtual CPU (vCPU) per tilt-image was used during pre-processing and 70 vCPUs per tilt-series for refinement. All runs used servers with dual Intel Xeon Gold 6154 and 768 GB of RAM.

DATASET	HIV-1 Gag (5 TS)	HIV-1 Gag (all TS)	80S in-vitro ribosome	70S in-cell ribosome	80S lamella ribosome	Mouse heavy chain apoferritin
**Raw data**						
EMPIAR ID	10164	10164	10064	10499	10987	11273
Detector	Gatan K2	Gatan K2	Gatan K2	Gatan K3	Gatan K3	Falcon 4i
Pixel size (Å)	0.675	0.675	2.62	1.7	1.64	0.729
Number of tilt-series	5	43	4	65	20	100
Tilt range	±60°	±60°	-56°, +58°	±60°	-51°,+69°	±48
Tilt spacing	3°	3°	2°	3°	3°	3°
Tilt images	41	41	61	41	41	33
Frames per tilt	8	8/10	1	1	5	153
**Data processing**						
Box size (voxels) | bin	384 | 2x	384 | 2x	256 | 1x	254 | 1x	300 | 1x	800 | 1x
Initial reference<br>*Lowpass filter (Å)*	From STA<br>N/A	From STA<br>N/A	From STA<br>N/A	EMD-11650<br>16	EMD-33118<br>16	EMD-11638<br>6
Symmetry imposed	C6	C6	C1	C1	C1	O
Initial particles	37,745	425,424	4,384	18,165	16,158	34,539
Final particles	14,482	109,496	4,384	18,165	6,763	31,890
Final 2D projections	4,287,532	35,545,312	231,210	740,800	234,815	5,169,905
Regions for refinement	8×8×2	8×8×2	8×8×2	1×1×1	4×4×2	4×4×2
Tilt-range for refinement	-6° to 6°	-6° to 6°	-38° to 22°	-30° to 27°	-15° to 30°	-6° to 6°
Number of classes	1	1	5	3	5	1
Map resolution (Å)<br>*0.143-cutoff FSC*	3.2	3.0	10.0/10.2/10.<br>2/10.0/10.0	8.7/9.3/9.5	12.4/11.8/11.<br>4/10.5/10.9	1.8
EMDB ID (s)	41196	41197	41205,41207,<br>41210,41211,<br>41212	41220,<br>41221,<br>41222,	41223,41224,<br>41225,41226,<br>41227	41199

Supplementary Table 3. Raw data and image processing statistics. Details of raw data from six EMPIAR datasets and parameters used for data processing. Corresponding FSC plots are presented in Fig. 4, Extended Data Figs. 2 and 5, and Supplementary Fig. 1.

OPERATION	DESCRIPTION	REFERENCES
**Movie frame alignment**		
Global alignment	Re-implementation based on cisTEM's unblur	[52], [54]
**Tilt-series alignment**		
Fiducial-based	Re-implementation using IMOD's `tiltxcorr` and RAPTOR	[24], [58]
Patch-based	Re-implementation using IMOD's `imodchopconts`	[24]
**CTF estimation**		
Tilted, astigmatic model	Re-implementation of methods used in BISECT	[13]
**Particle picking**		
Geometry-based	Re-implementation of previous methods	[31], [42]
Neural network-based	Re-implementation of previous methods	[45]
Size-based	New algorithm for 3D particle picking	This work
**Refinement**[1]		
Stack-less framework	New scalable architecture for constrained SP-CET	This work
Reference-based	New particle alignment strategy using 2D projections	This work
Fully constrained	New implementation of constrained SP-CET refinement	This work[2]
Region-based	New implementation of constrained SP-CET refinement mode	This work[2]
Per-particle CTF	New implementation of constrained SP-CET refinement mode	This work[2]
Movie frames	New constrained SP-CET refinement mode	This work
**Classification**[1]		
Constrained classification	New 3D classification method	This work

[1] All operations are implemented using cisTEM primitives
[2] Similar strategies using Relion primitives were introduced in M

Supplementary Table 4. List of re-implementations and new methods used in nextPYP. nextPYP combines re-implementations of existing algorithms with newly introduced methods that together provide a scalable and end-to-end framework for constrained SP-CET image analysis.

	Size based picking	Neural network based picking
**EMPIAR-10304 (in vitro)**		
Average particles per tilt-series	1,074	1,341
Precision	**0.71**	0.70
Recall	0.65	**0.76**
F1	0.68	**0.73**
**EMPIAR-10499 (in situ)**		
Average articles per tilt-series	476	555
Precision	0.52	**0.61**
Recall	0.55	**0.74**
F1	0.54	**0.67**

Supplementary Table 5. Comparison between size based and neural network based particle picking. Precision, recall and F1 scores are presented for each method using tomograms from two datasets of ribosomes: EMPIAR-10304 (in vitro), and EMPIAR-10499 (in-situ). Manually picked particles were used as ground truth to calculate all the accuracy metrics. For the neural network based approach, 230 particles were used during training for the EMPIAR-10304 dataset, and 195 particles for EMPIAR-10499 tilt-series.
